# Electroacupuncture Improves Synaptic Function in SAMP8 Mice Probably via Inhibition of the AMPK/eEF2K/eEF2 Signaling Pathway

**DOI:** 10.1155/2019/8260815

**Published:** 2019-09-18

**Authors:** Weiguo Dong, Wendan Yang, Feifei Li, Wanqing Guo, Changhui Qian, Feng Wang, Changzhen Li, Lan Lin, Ruhui Lin

**Affiliations:** ^1^Department of Integrated Traditional Chinese and Western Medicine, Fujian University of Traditional Chinese Medicine, Fuzhou 350122, China; ^2^Fujian Key Laboratory of Rehabilitation Technology, Fuzhou 350122, China; ^3^Department of Acupuncture and Moxibustion, Fujian University of Traditional Chinese Medicine, Fuzhou 350122, China; ^4^The Third People's Hospital of Fujian Province, Fuzhou 350108, China; ^5^Academy of Integrative Medicine, Fujian University of Traditional Chinese Medicine, Fuzhou 350122, China

## Abstract

Synaptic loss and dysfunction is associated with cognitive impairment in Alzheimer's disease (AD). Recent evidence indicates that the AMP-activated protein kinase (AMPK)/eukaryotic elongation factor-2 kinase (eEF2K)/eukaryotic elongation factor-2 (eEF2) pathway was implicated in synaptic plasticity in AD. Therapeutic strategies for AD treatment are currently limited. Here, we investigate the effects of electroacupuncture (EA) on synaptic function and the AMPK/eEF2K/eEF2 signaling pathway in male senescence-accelerated mouse-prone 8 (SAMP8) mice. Male 7-month-old SAMP8 and SAMR1 mice (senescence-accelerated mouse resistant 1) were randomly divided into 3 groups: SAMR1 control group (Rc), SAMP8 control group (Pc), and SAMP8 electroacupuncture group (Pe). The Pe group was treated with EA for 30 days. Transmission electron microscopy (TEM) was used to observe the structure of synapse. The protein and mRNA expression of synaptophysin (SYN) and postsynaptic density 95 (PSD95) was examined by immunohistochemistry, western blot, and real-time RT-PCR. The activity of AMPK and eEF2K was studied by western blot. Our results showed that EA ameliorated synaptic loss, increased the expression of SYN and PSD95, and inhibited AMPK activation and eEF2K activity. Collectively, these findings suggested that the mechanisms of EA improving synaptic function in AD may be associated with the inhibition of the AMPK/eEF2K/eEF2 signaling pathway.

## 1. Introduction

Alzheimer's disease (AD) is the most prevalent neurodegenerative disease characterized by the presence of extracellular amyloid plaque deposits and the intraneuronal neurofibrillary tangles (NFTs) in the brain [1]. In addition to amyloid plaques and NFTs, synaptic failure is an early event in AD pathogenesis [2–4] and correlates best with cognitive deficits in AD [5, 6]. Furthermore, amyloid *β*-protein (A*β*) and tau protein cause synaptic dysfunction [7–9]. Thus, targeting synaptic dysfunction has been proposed as a potential therapeutic approach to rescue cognitive dysfunction in AD [10, 11].

AMPK is a heterotrimeric protein composed of a catalytic *α* subunit and regulatory *β* and *γ* subunits [12], often referred to as an important sensor of cellular energy status. It has been reported that AMPK activity, as evaluated by phosphorylation of the *α* subunit at Thr172, is significantly elevated in human AD brains and AD animal models [13–16]. In AD, AMPK was involved in A*β* production and tau pathology [17, 18] and mediated the toxic effects of A*β* on synapses [14, 15]. In addition, AMPK hyperactivation induced synaptic loss in primary neuronal cultures [19].

Eukaryotic elongation factor-2 kinase (eEF2K) is a member of the calcium-/calmodulin-dependent kinase family, which couples cellular energy status to protein synthesis [20]. eEF2K has been shown to be activated by AMPK [21–24]. Activated eEF2K phosphorylates the eukaryotic elongation factor-2 (eEF2) on threonine-56 (Thr56) residue [25]. In addition to regulating energy homeostasis, eEF2K/eEF2 pathway has also been implicated in synaptic plasticity and A*β*-induced synaptic dysfunction in AD [15, 26, 27]. Recent studies indicate that eEF2K activity is upregulated in the cortex and hippocampus of AD patients and in the hippocampus of transgenic AD mice [15, 28, 29]. Conversely, inhibiting eEF2K alleviates synaptic dysfunction in AD [15, 29, 30]. In addition, AMPK/eEF2K/eEF2 pathway is associated with A*β*-induced synaptic deficits [15].

Both clinical studies and animal studies have shown that electroacupuncture (EA) is a potential therapy for AD [31–34]. Recent studies have suggested that EA improves synaptic plasticity in animal models of AD [35, 36]. However, the underlying mechanisms of EA treatment for improving synaptic plasticity of AD are not completely understood. We recently showed that EA treatment could effectively improve cognitive deficits as well as AMPK activity in the hippocampus of SAMP8 mice [37, 38]. In addition, the hippocampus, one of the brain areas involved in memory [39], is one of the regions most affected by neurodegeneration in AD. In this research, we focused on the hippocampal CA1 area because of the critical role in memory processing and the significant functional, structural, and morphological alterations in AD [40]. In the present study, we investigate the effects of EA on synaptic function and AMPK/eEF2K/eEF2 pathway. Our study may provide novel findings about the mechanisms of EA improving synaptic function in AD.

## 2. Materials and Methods

### 2.1. Animals

Three-month-old male senescence-accelerated mouse-prone 8 (SAMP8) and senescence-accelerated mouse resistant 1 (SAMR1) mice were obtained from the Department of Laboratory Animal Science of Peking University. Mice were housed in a 12 h light/dark cycle at constant temperature with free access to food and water. All animal procedures were approved by the Ethics Committee for Laboratory Animals at Fujian University of Traditional Chinese Medicine. When the mice were aged 7 months, the experiment was initiated. 42 male SAMP8 were randomly divided into SAMP8 control group (Pc) and SAMP8 electroacupuncture group (Pe). 21 male homologous SAMR1 mice were used as normal control group (Rc). Mice in the Pe group received EA treatment. No treatment was carried out in the Rc and Pc groups, with grabbing and fixing the mice in order to ensure the same treatment conditions as that in the Pe group.

### 2.2. EA Treatment

**Figure 1:**
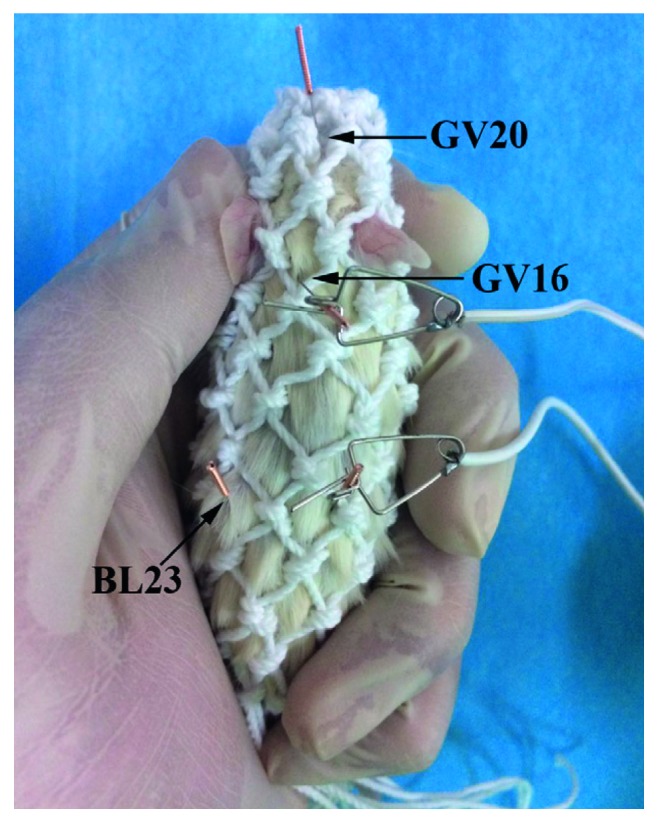
Schematic diagram of mouse immobilization and acupoints of Baihui (GV20), Dazhui (GV16), and Shenshu (BL23).

EA treatment was performed as described in our previous study [37]. Briefly, we used nets to fix the mice by an assistant's hands during the entire treatment (Figure 1). Four stainless steel acupuncture needles were inserted at a depth of 5 mm into the “Baihui” (GV20, at the midpoint between the auricular apices), “Dazhui” (GV14, below the spinous process of the seventh cervical vertebra), and bilateral “Shenshu” (BL23, at the depression lateral to the lower border of spinous process of the second lumbar vertebra) acupoints. Acupuncture needles were separately inserted horizontally downward at GV20 and perpendicularly at GV14 and at BL23. The needles at GV14 and the side BL23 were connected to the output terminals of the EA instrument (Hwato, model no. SDZ-V, Suzhou Medical Instruments Co., Ltd., Suzhou, China). BL23 was alternately stimulated on the left and right sides every day. We performed continuous-wave stimulation at a frequency of 2 Hz (intensity 1 mA). An individual EA session was administered daily for 20 min, 8 days, and 2 days of rest, for a period of 30 days.

### 2.3. Transmission Electron Microscopy (TEM)

Three mice in each group were used for electron microscopy. In brief, the mice were transcardially perfused with 4% paraformaldehyde and 1% glutaraldehyde in 0.1 M phosphate buffer. The removed hippocampal CA1 area was fixed in 2.5% glutaraldehyde at 4°C for 2 h and later fixed with 1% osmium tetroxide (2 h, 4°C). The samples were dehydrated in a graded series of acetone (50%, 70%, 90%, and 100%) and embedded in Epon resin at room temperature for 24 h. Series of 90 nm ultrathin sections were cut with a 35° diamond knife (Diatome) on a Reichert ultracut E ultramicrotome (Leica) and mounted on Formvar-coated grids. The sections were stained with uranyl acetate and lead acetate. Micrographs were observed and photographed using a HITACHI H-7650 transmission electron microscope (HITACHI, Japan). Fifteen fields of the hippocampal CA1 area of the mice from the Rc, Pc, and Pe groups (five fields per mouse from a total of three mice from each group) were chosen randomly and examined. The number of synapses and the thickness of PSD in hippocampus CA1 were analyzed using Image Pro Plus 6.0.

### 2.4. Immunohistochemistry

Immunohistochemistry was performed as described previously [37]. Six mice from each group were anesthetized and transcardially perfused with 0.9% saline followed by 4% paraformaldehyde in PBS. The brains were removed and fixed in 4% paraformaldehyde/PBS overnight at 4°C. The tissue blocks containing the hippocampus were dehydrated and embedded in paraffin. The 5 *μ*m thick serial sections were cut in a coronal plane under a Leica microtome (Leica RM 2135) and mounted on 0.1% polylysine reagent-coated slides (Sigma). Then, rehydrated and ethanol-cleared sections were incubated with 3% H_2_O_2_ for 15 min at room temperature to block the endogenous peroxidase reaction and rinsed 3 × 10 min in PBS. Slides were treated with microwave (700 W) in 0.05 mol/L citrate-buffered saline (pH 6.0) for 10 min for antigen retrieval and washed in PBS for 3 × 10 min. Then, the sections were incubated in 5% normal goat serum for 30 min to block nonspecific antibody binding. The sections were next incubated with rabbit anti-synaptophysin (SYN) antibody (1 : 200, Abcam 32127) and rabbit anti-postsynaptic density 95 (PSD95) (1 : 200, Abcam 18258) overnight at 4°C. The sections were then incubated with biotinylated goat anti-rabbit IgG secondary antibodies (1 : 200, Vector Laboratories, Inc.). The signal was visualized using a diaminobenzidine (DAB) kit (Vector Laboratories, Burlingame, USA) according to the manufacturer's instructions. Images were digitized using a microscope (BX-51; Olympus) and analyzed by Image J software. The measurement was performed by two researchers blinded to the treatment condition.

### 2.5. Western Blot Analysis

Six mice from each group were used for the western blot. Lysates were prepared as previously described [38]. Equal amounts of protein (25 *μ*g) from each sample were separated by Tris-glycine SDS-PAGE and electrophoretically transferred onto nitrocellulose membranes (Millipore) on ice. Membranes were blocked for 1 h at room temperature with blocking buffer (5% nonfat dry milk in Tris-buffered saline with 0.05% Tween 20 (TBS-T)) and then incubated overnight at 4°C with the following primary antibodies (at dilutions of 1 : 1000, unless otherwise specified): anti-SYN (Abcam 32127), PSD95 (Abcam 18258), P-AMPK*α* (T172) (Cell Signaling Technology 2535), AMPK*α* (Cell Signaling Technology 2532), p-eEF2 Thr56 (Cell Signaling Technology 2331), eEF2 (Cell Signaling Technology 2332), and rabbit anti-*β*-actin (1 : 10,000, Sigma). After several washes, the membranes were incubated with the appropriate HRP-conjugated secondary antibodies at room temperature for 2 h, and then, the protein signal was visualized using an enhanced chemiluminescence detection kit (Thermo). Bands were scanned using the FluorChem scanner and quantified with the NIH Image J software. These results were normalized with *β*-actin expression levels and confirmed by triplicate measurements of the same sample.

### 2.6. Real-Time RT-PCR

RNA samples were extracted from the mouse hippocampus using the RNeasy Mini Kit (Qiagen, Valencia, CA, USA) according to the manufacturer's instructions. Reverse transcription was performed on 1 *μ*g of total RNA using the High Capacity cDNA Reverse Transcription Kit (Applied Biosystems). The synthesized cDNA was stored at −80°C for further use. Quantitative PCR was performed with Power SYBR Green PCR Master Mix (Applied Biosystems) on a 7300 real-time PCR system (Applied Biosystems) using the default thermal cycling program. The following primers were used: SYN (forward 5′-CTGCGTTAAAGGGGGCACTA-3′ and reverse 5′-ACAGCCACGGTGACAAAGAA-3′), PSD95 (forward 5′-CTTCATCCTTGCTGGGGGTC-3′ and reverse 5′-TTGCGGAGGTCAACACCATT-3′), and *β*-actin (forward 5′-AGAAGCTGTGCTATGTTGCTCTA-3′ and reverse 5′-TCAGGCAGCTCATAGCTCTTC-3′) [41]. The data were analyzed by the relative ΔΔCT quantification method using *β*-actin CT values as internal reference in each sample.

### 2.7. Statistical Analysis

Data were tested for normal distribution using the Kolmogorov*–*Smirnov test. Normally distributed data are shown as the mean ± standard deviation and were analyzed using one-way ANOVA. A one-way ANOVA with a least significant difference test or a one-way ANOVA with post hoc Dunnett's T3 was performed to compare between groups. Significance was set at *p* < 0.05.

## 3. Results

### 3.1. EA Ameliorated Synaptic Loss and Increased PSD Thickness in SAMP8 Mice

**Figure 2:**
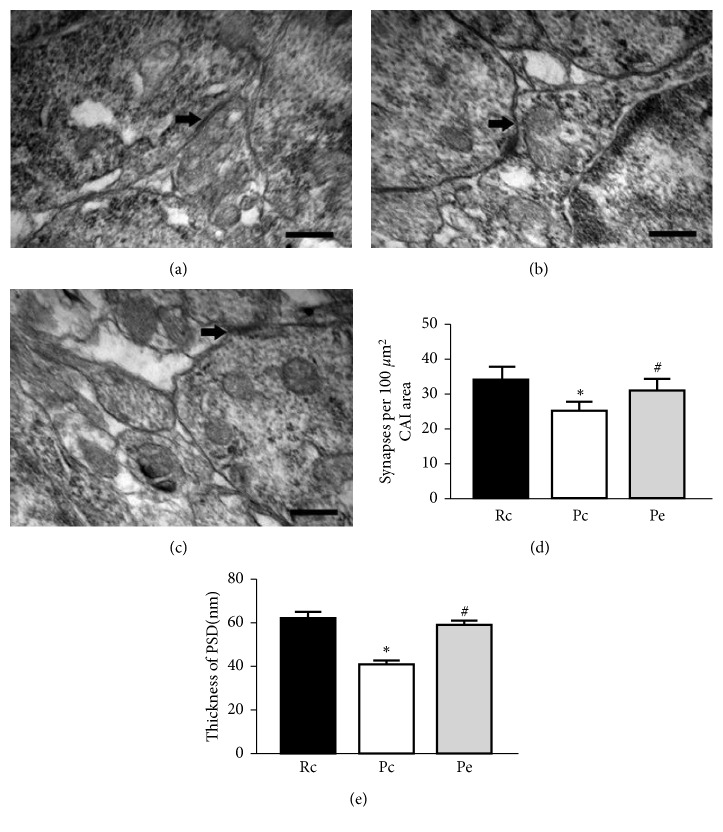
Effects of EA on the number of synapses and the thickness of PSD in the hippocampal CA1. Representative electron microscopy of the synaptic structures in the hippocampal CA1 area in Rc (a), Pc (b), and Pe (c). Arrows indicate the synapses, scale bar 100 nm. (d) Quantitative analysis of the synaptic density in Rc, Pc, and Pc groups. (e) The quantitative comparison of the PSD thickness in Rc, Pc, and Pc groups. ^*∗*^*p* < 0.05, compared with the Rc group. ^#^*p* < 0.05 when compared with the Pc group.

As shown in Figure 2, the number of synapses and the thickness of PSD in the Pe group were increased compared with the Pc group. The number of synapses and the thickness of PSD in the Pc group were decreased when compared with those detected in the Rc group. No statistically significant difference was found between the Rc and Pe groups (*p* > 0.05).

### 3.2. EA Upregulated the mRNA and Protein Levels of SYN and PSD95 in SAMP8 Mice

**Figure 3:**
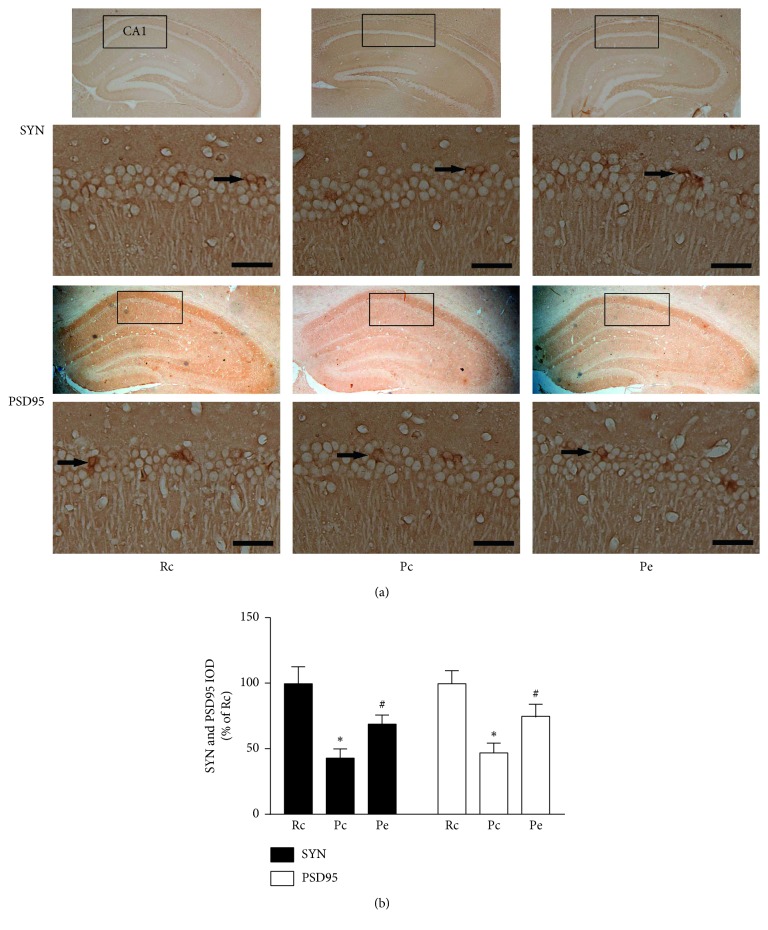
Immunohistochemical positive expression of SYN and PSD95. (a) Representative immunohistochemical stainings for SYN and PSD95 positive areas in the hippocampal CA1 area. Black arrows show the hippocampal CA1 area-positive staining. Scale bar 50 *μ*m. (b) Quantification of integrated optical density (IOD) by immunoreactivity to SYN and PSD95. ^*∗*^*p* < 0.05, compared with the Rc group. ^#^*p* < 0.05 when compared with the Pc group.

Some of the proteins most often reported to evaluate synaptic function are SYN (a presynaptic marker protein) and PSD95 (a postsynaptic marker protein). Representative photomicrographs of the immunohistochemical staining demonstrated brownish yellow granules in pyramidal cells in the hippocampal CA1 areas (Figure 3(a)). As shown in Figure 3(b), the integrated optical density (IOD) of SYN and PSD95 immunostaining was significantly decreased in the Pc group compared with the Rc group. The IOD in the Pe group were higher than that in the Pc group. There were no significant differences in IOD between the Pe group and the Rc group.

**Figure 4:**
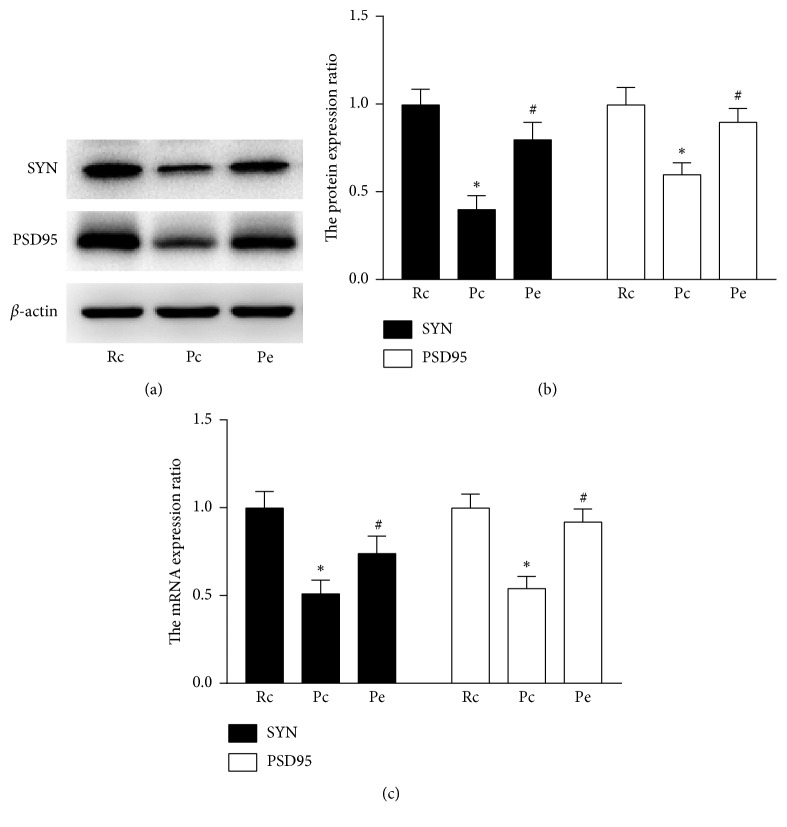
Protein and mRNA expression of SYN and PSD95. (a) Representative western blot of SYN and PSD95 in the hippocampus of Rc, Pc, and Pe groups. (b) Quantification of SYN and PSD95 protein expression. (c) Analysis of relative mRNA level of SYN and PSD95 by qRT-PCR using 2^−ΔΔCt^. ^*∗*^*p* < 0.05, compared with the Rc group. ^#^*p* < 0.05, compared with the Pc group.

Consistent with the immunohistochemical results, immunoblots (Figure 4(a)) and relative protein expression analyses (Figure 4(b)) showed that the SYN and PSD95 protein levels in the Pc group were significantly decreased in comparison to the Rc group, but EA increased the expression of SYN and PSD95 compared with the Pc group, and there were no significant differences between the Pe and Rc groups.

The qRT-PCR results were consistent with the evidences from immunohistochemistry and western blot. As shown in Figure 4(c), compared to the Rc group, the mRNA levels of SYN and PSD95 were decreased in the Pc group, but the mRNA levels of SYN and PSD95 in the Pe group were increased when compared to the Pc group, and there were no significant differences between the Pe and Rc groups.

The present results demonstrated that the mRNA and protein expression of synaptic protein markers (SYN and PSD95) is decreased in SAMP8 mice and EA upregulates the transcription and protein expression of synaptic proteins.

### 3.3. EA-Suppressed AMPK Phosphorylation in SAMP8 Mice

**Figure 5:**
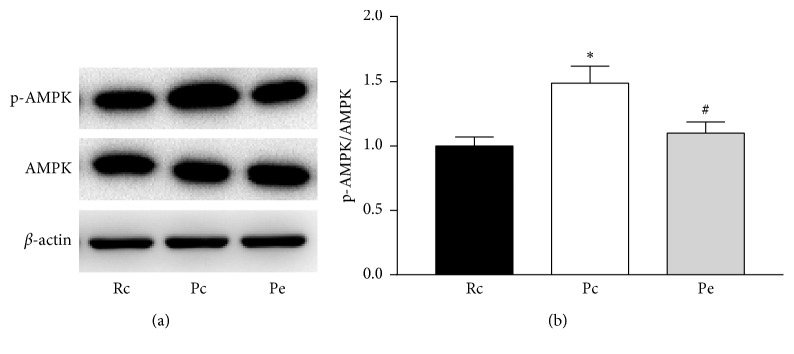
EA-suppressed AMPK phosphorylation in SAMP8 mice. AMPK phosphorylation (Thr172) in the hippocampus of Rc, Pc, and Pe groups are shown by western blot experiments (a) and the quantification graph (b). ^*∗*^*p* < 0.05 versus Rc group. ^#^*p* < 0.05 versus Pc group.

We investigated the effect of EA on AMPK activity in SAMP8 mice by measuring phosphorylation at the Thr172 site on the *α*-subunit of AMPK. We observed an elevated phosphorylation of AMPK in the Pc group compared with the Rc group, and EA treatment suppressed AMPK phosphorylation compared with the Pc group, whereas there were no significant differences between the Pe and Rc groups (Figure 5).

### 3.4. EA-Inhibited eEF2K Activity in SAMP8 Mice

**Figure 6:**
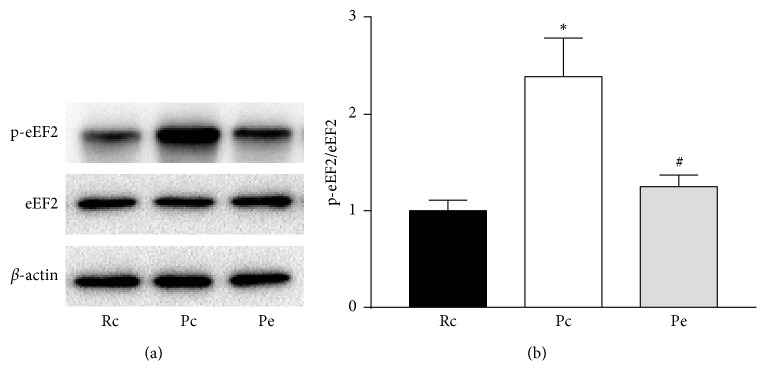
EA-suppressed eEF2K activity in SAMP8 mice. (a) Representative blots for p-eEF2 and eEF2, from the hippocampus of Rc, Pc, and Pe groups. (b) Quantification of western blots for p-eEF2/eEF2. ^*∗*^*p* < 0.05 versus Rc group. ^#^*p* < 0.05 versus Pc group.

Previous studies have revealed that the eEF2K/eEF2 pathway is involved in synaptic plasticity in AD [15]. The levels of phosphorylated eEF2 reflect eEF2K activity [29]. Thus, we further examined the effect of EA on the eEF2K/eEF2 pathway in SAMP8 mice by measuring eEF2 phosphorylation. Western blot for p-eEF2 showed that the levels of p-eEF2 were higher in the Pc group compared with the Rc group, and the levels of p-eEF2 in the Pe group were decreased compared with the Pc group, whereas there were no significant differences between the Pe and Rc groups (Figure 6).

## 4. Discussion

Accumulating evidence suggests that synaptic loss and dysfunction may be the major causes of early AD development [2–4]. Thus, reversing synaptic dysfunction has been considered as a promising therapeutic approach for the treatment of AD [42]. The present study demonstrates that EA treatment increases synaptic loss and PSD thickness and upregulates the expression of synaptic-related protein (SYN and PSD95) in SAMP8 mice, an animal model of sporadic AD [43–45]. SAMR1, which has a genetic background similar to that of SAMP8, does not exhibit senescence-related neuronal phenotypes and has been used extensively as a control strain [46]. In addition, we show that EA treatment suppresses the AMPK/eEF2K/eEF2 signaling pathway, which is implicated in AD-related synaptic pathophysiology [15]. Taken together, these results suggest that EA treatment can improve synaptic function probably via inhibition of the AMPK/eEF2K/eEF2 signaling pathway.

AMPK is an important energy sensor and is overactivated in AD brains [13, 15]. However, the exact role played by AMPK in AD remains to be clearly established. In the current study, we observed that AMPK activity was increased in 8-month-old SAMP8 mice compared with age-matched SAMR1 mice. This observation is in contrast with our previous report showing that AMPK phosphorylation is increased in 5-month-old SAMP8 mice compared with age-matched SAMR1 mice [37]. This discrepancy may be attributed to different ages of mice used in two studies.

It has been shown previously that AMPK activation might be beneficial at the onset of AD, whereas chronic AMPK activation becomes detrimental for neurons in the late stages of AD [47]. Thus, increased AMPK activity at the onset of AD and inhibition of AMPK activity in the late stages of AD provide protective effects. Consistent with this notion, we have previously shown that EA increases AMPK activity in 4-month-old SAMP8 mice. We now show that EA inhibits AMPK activity in 7-month-old SAMP8 mice. On the contrary, these results exhibit the bidirectional benign adjustable action of acupuncture.

According to the Traditional Chinese Medicine theory, the kidney essence deficiency and the marrow sea deficiency are the main causes of AD, and brain is the sea of marrow [48]. The BL23 collects the kidney essence and marrow, which is the origin of congenital constitution. It has been reported that acupuncture at BL23 could improve cognitive deficits in animal models of AD [48, 49]. In addition, Traditional Chinese Medicine holds the theory that the governor vessel (GV) collects the yang and transports the yang to the brain. Thus, the GV is closely associated with brain function. GV20 and GV14 both belong to the GV. It has been demonstrated that EA stimulation at GV20 could improve cognitive deficits in APP/PS1 mice [34, 50–52]. Furthermore, acupuncture or EA at the GV20 and BL23 acupoints has been shown to improve memory in AD model rats [49, 53, 54]. We previously reported that EA stimulation at the GV14 and BL23 acupoints ameliorates cognitive impairment in SAMP8 mice [37, 38, 55]. On the basis of the above theories and results, we chose GV20, GV14, and BL23 to treat AD mice in this study.

EA has demonstrated an effective therapeutic intervention for AD in a large number of animal studies [33]. Our previous studies also showed that EA improved cognitive deficits in SAMP8 mice [37, 38, 55]. Cognitive impairment in AD is attributable to disruptions of synaptic functions which correlate with the severity of memory deficit in AD [2, 5, 6]. EA has been shown to enhance hippocampal long-term potentiation (LTP) in AD animal models [35, 36]. Therefore, we focused on the effects of EA on synapses in AD. SYN, a presynaptic vesicle protein, regulates synaptic plasticity and synapse formation [56, 57]. PSD95 plays a critical role in synaptogenesis and synaptic plasticity [58]. Our study showed that EA increased mRNA and protein expression of SYN and PSD95 in the hippocampus of SAMP8 mice. Additionally, we further demonstrated that EA improved the structures of synapses in the hippocampal CA1 area of SAMP8 mice. These results indicate that EA can alleviate hippocampal synaptic dysfunction in AD. However, the underlying mechanisms of EA treatment for improving synaptic function of AD are not well known.

eEF2K is a key molecule that couples cellular energy status to protein synthesis [20]. Consistent with previous studies [15, 28, 29], our study showed that eEF2K activity was increased in the hippocampus of SAMP8 mice compared to SAMR1 mice. Furthermore, eEF2K/eEF2 pathway is associated with synaptic plasticity [26, 59–61]. Inhibition of eEF2K/eEF2 pathway prevents synaptic failure in AD [29, 30]. AMPK is an upstream regulator of eEF2K, and AMPK activation leads to increase eEF2 phosphorylation by direct activation of eEF2K [24]. Inhibition of AMPK/eEF2K/eEF2 pathway reduces AD-related synaptic dysfunction [15]. Here, we show that EA suppresses the AMPK/eEF2K/eEF2 pathway in SAMP8 mice. Hence, it is plausible that EA treatment for improving synaptic function of AD is mediated by inhibiting the AMPK/eEF2K/eEF2 pathway.

## 5. Conclusions

The present findings show that EA at GV20, GV14, and BL23 improves synaptic function in SAMP8 mice. We propose that the underlying mechanism of EA for improving synaptic function of AD may be related to the inhibition of AMPK/eEF2K/eEF2 pathway in the hippocampus. In the future, we will further evaluate the effects of EA on synapses in AD model mice under the condition of the inhibition or activation of AMPK and eEF2K activities in CA1 area of the hippocampus and other areas of the hippocampus and the cerebral cortex, respectively.

## Data Availability

The data used to support the findings of this study are available from the corresponding author upon request.
